# Ligation of the mesovarium in dogs with a self-locking implant of a resorbable polyglycolic based co-polymer: a study of feasibility and comparison to suture ligation

**DOI:** 10.1186/s13104-016-2042-2

**Published:** 2016-04-27

**Authors:** Matheus Roberto da Mota Costa, André Lacerda de Abreu Oliveira, Renato Moran Ramos, Leonardo Waldstein de Moura Vidal, Niklas Borg, Odd V. Höglund

**Affiliations:** Department of Animal Clinical and Surgery, Universidade Estadual Norte Fluminense, UENF/RJ, Av. Alberto Lamego, 2000, Campos dos Goytacazes, RJ Brazil; Department of Veterinary Surgery and Anaesthesia, Universidade Iguaçu, UNIG/RJ, BR-356/KM 02, Itaperuna, RJ Brazil; Resorbable Devices AB, Ormvråksvägen 15, SE-756 52 Uppsala, Sweden; Department of Clinical Sciences, Swedish University of Agricultural Sciences, Box 7054, SE-750 07 Uppsala, Sweden

**Keywords:** Medical device, Glycolide, Trimethylene carbonate, Ovarian pedicle, Canine, Ovariohysterectomy, Spay

## Abstract

**Background:**

Ligation of the mesovarium in female dogs may be cumbersome with risk of complications and is associated with intense noxious stimuli. A resorbable implant, a self-locking loop designed for surgery, was developed as an alternative to traditional ligation. The study aimed to test the feasibility of ligating the canine ovarian pedicle with the implant and to compare its performance to traditional suture ligation.

**Results:**

In total 45 intact female dogs destined for elective ovariohysterectomy and adoption were included. In 21 dogs the new resorbable implant was used to ligate the mesovarium, and in 24 control dogs traditional suture was used with one encircling ligature. Mean weight of implant dogs was 10.7 ± 5.6 kg (range 3.5–22.0), and mean weight of control dogs was 12.8 ± 6.4 kg, (range 4.1–27.0). The body weight of dogs did not differ between groups (P = 0.25). In total, 42 ovarian pedicles were successfully ligated with the implant. In one control dog, intraoperative haemorrhage from the left ovarian pedicle was diagnosed. The mesovarium was re-ligated and haemostasis was confirmed. All dogs recovered uneventfully. The ligation time of the mesovarium was significantly shortened (P = 0.02) by using the self-locking implant versus a single ligature (3′28′′ ± 1′05′′ and 5′29′′ ± 3′54′′, respectively). Total duration of surgery differed between the groups (P = 0.02) with a shortened duration of surgery when using the self-locking implant (15′56′′ ± 2′47′′ and 20′39′′ ± 8′58′′, study group versus control group, respectively). In both groups, duration of surgery and time required to ligate the ovarian pedicle were longer in larger dogs than smaller dogs.

**Conclusion:**

The results of this feasibility study suggested the implant can be used to ligate the canine mesovarium. Compared with traditional suture ligation, the results suggested that time to ligate the ovarian pedicle and duration of surgery were significantly reduced with the implant. More time was required to perform surgeries in larger dogs.

## Background

The surgical procedure to desex female dogs involves ovaries removal. One intra- or postoperative complication is haemorrhage from the ovarian pedicle [1] which occurs more often in large and deep-chested breeds [2]. Moreover, duration of surgery is longer in larger dogs [3]. Furthermore, ovaries removal is a part of the surgery with intense noxious stimuli, which causes a surgical stress response [4–7]. This calls for new and improved ligation techniques.

A cable tie or tie rap, a self-locking loop, is a flexible band with a locking case at the end. Cable ties became popular in veterinary surgery in the 1970s. It is suggested that duration of a bitch spay surgery can be reduced if self-locking loops are used to ligate the mesovarium [8–11]. However, traditional cable ties are not designed for surgery, are made without declared good manufacturing practice (GMP) and can contain substances not suited for long term implantation. The material is nylon, a non-resorbable material which may cause chronic tissue reactions [12–15]. Therefore the use of traditional cable ties in surgery is strongly discouraged [16], as is the use of non-resorbable material for ligation purposes [17–20].

In a university research project a self-locking loop designed for surgical ligation was developed. It was first manufactured in the resorbable polymer polydioxanone [21–23] and later in a resorbable block co-polymer of glycolide (GA) and trimethylene carbonate (TMC) [24, 25] equivalent to the resorbable suture Maxon™ [26].

The aim of this study was to test the feasibility of ligating the canine ovarian pedicle with the self-locking implant made of the resorbable block co-polymer of GA and TMC and to compare its performance to that of traditional suture ligation.

## Methods

### Animals

A prospective clinical trial was performed to test the medical device. In total 45 privately owned or “spay-and-adopt” intact females destined for elective ovariohysterectomy and adoption were included in the study (Table 1). In 21 dogs the new resorbable implant was used to ligate of the mesovarium, and in 24 dogs traditional suture was used as control. Mean body weight ± standard deviation of implant dogs was 10.7 ± 5.6 kg (range 3.5–22.0), and that of control dogs was 12.8 ± 6.4 kg, (range 4.1–27.0).

**Table 1:** Description of weight and estimated age of the dogs

Groups	Weight (kg)	Age (years)	N
Implant–LigaTie^®^	10.7 ± 5.6	4.1 ± 2.2	21
Control—suture ligation	12.8 ± 6.4	3.5 ± 2.1	24

Data are mean ± SD

Before surgery all dogs were microchipped and underwent a full clinical examination, including a complete haemogram. Surgery was done on the control group first due to delayed delivery of implants. An informed consent was obtained from the owners before inclusion of their dog in the study. The study protocol was approved by the local ethical committee, reference UENF-CEUA 435813.

### Anaesthesia

Dogs were premedicated with acepromazine, tramadol and meloxicam (Table 2). Anaesthesia was induced and maintained with ketamine and midazolam, after which epidural anaesthesia was administered with lidocaine.

**Table 2:** Drugs used for premedication and anaesthesia of the 45 dogs

Drug	Indication	Dosage (mg/kg)	Trade name	Route of administration
Acepromazine	Sedative	0.1	Apromazin 0.2 %^a^	I.M.
Tramadol	Analgesic	4	Cloridrato de tramadol 50 mg/ml^b^	I.M.
Meloxicam	Analgesic	0.2	Maxicam 2 %^c^	S.C
Ketamine	General anaesthesia	10	Cetamin 10 %^a^	I.V.
Midazolam		0.5	Midazolam 5 mg/ml^b^	I.V.
Lidocaine	Regional anaesthesia	5	Xylestesin 2 %^d^	Epidural

^a^ Syntec^®^

^b^ Hipolabor

^c^ Ouro Fino

^d^ Cristália

### The resorbable implant

**Fig. 1:**
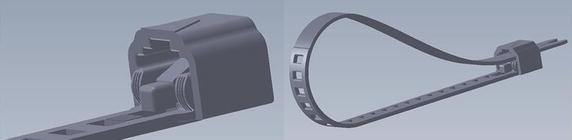
The resorbable implant, LigaTie^®^, was constructed as a flexible band and case with a locking mechanism. Design features for tissue engaging properties were added to the locking case to increase friction between implant and tissue (*left*). The band was introduced into the locking case, which allowed one-way motion only when the locking mechanism engaged with the perforations, and a self-locking loop was formed (*right*)

To construct the device computer-aided design (SolidWorks^®^, Dassault Systèmes SolidWorks Corporation, Concord, USA) was used. The implant (LigaTie^®^, legal manufacturer Resorbable Devices AB, Uppsala, Sweden, http://www.LigaTie.com) consisted of a flexible band, in part perforated, and a case with a locking mechanism where the band could be introduced and pulled through (Fig. 1). One-way motion only was allowed when the locking mechanism engaged with the perforations of the flexible band, and a self-locking loop was formed. Design features for tissue engaging properties, aimed to achieve a secured tissue grip, were added to the locking case to increase friction between implant and tissue that was compressed inside loop of implant.

Glycolide and TMC were polymerized into a block co-polymer. The resorbable polymer was injection moulded into LigaTie^®^ products. The produced devices were placed in Tyvek bags, two implants per bag. The Tyvek bags were sealed in a clean room, placed in aluminium foil pouches and allowed to dry under vacuum for at least 7 days. Pouches were then sealed in a moisture free environment. Implants were sterilized while inside the sealed aluminium foil pouches by using electron beam radiation at a dose of 25 kGy.

### Surgery

All operations were performed by the same surgeon, with sterile assistant, and followed standard procedures, except that the device was used to ligate of the ovarian pedicles and in the control group traditional suture of the equivalent material was used (Maxon™, size 0, 3.5 metric, monofilament polyglyconate synthetic absorbable suture, manufactured by Syneture, 150 Glover Avenue, Norwalk, Connecticut 06856 USA). Total surgery time and ligation time was recorded. Any observed intraoperative or postoperative bleeding was noted in a protocol.

**Fig. 2:**
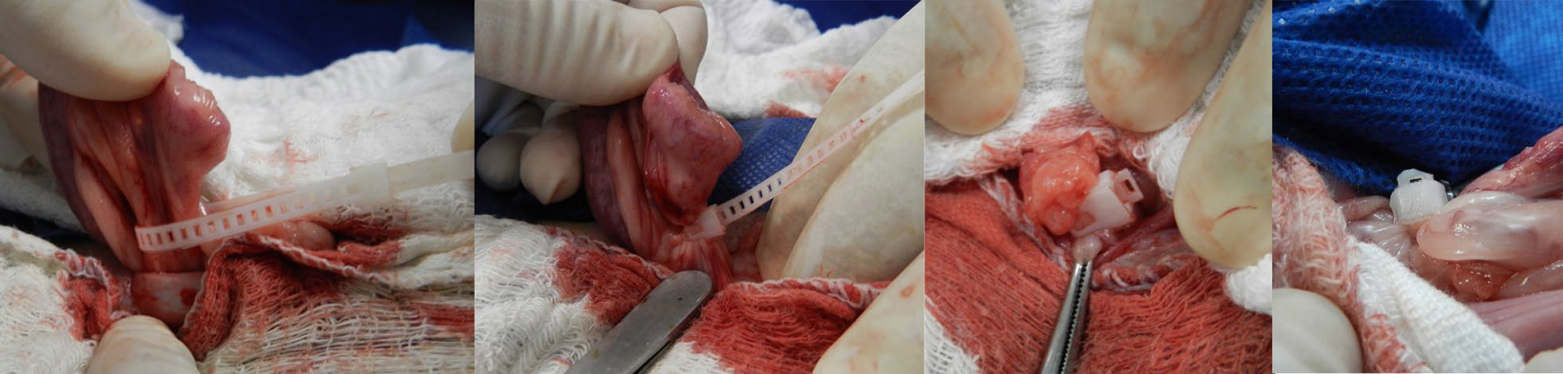
The self-locking loop of the implant was placed around the ovarian pedicle and tightened. Tissue was transected and after haemostasis was verified the excess band was cut off. In some cases care was taken to produce a rounded cut-off end (*far right*)

Dogs were placed in the Trendelenburg position and an incision was made along the midline. Uterine horns and ovaries were localized manually. A hole was made in the broad ligament close to the ovary. The flexible band was applied around the mesovarium (ovarian pedicle) and introduced into the locking case thus forming a loop (Fig. 2). The implant’s loop around the ovarian pedicle was tightened with one hand while the other hand secured the ovary. The tissue and blood vessels within the loop were compressed. A needle holder was occasionally used for improved grip of the flexible band of the implant at final check to ensure the loop was fully tightened.

After the loop of the implant was tightened and desired compression of tissue was achieved, a pair of forceps were used in-between the implant and the ovary. The ovarian pedicle was transected distal to the implant, close to the forceps. The tissue was inspected for haemorrhage to verify haemostasis. The procedure was repeated on the opposite side.

After haemostasis was verified a second time on the ovarian pedicle, excess band extending from the locking case was removed. A short segment of the flexible band remained protruding from the locking case to allow grabbing the flexible band with a needle holder and to enable further tightening of the loop, if needed. The procedure was repeated on the opposite side.

In control dogs, a pair of haemostatic forceps was used to clamp the ovarian pedicles and make a groove. The forceps were then repositioned distally, closer to the ovary and one ligature was placed in the compressed tissue. An extra clamp was placed on the suspensory ligament. The ovarian pedicle was cut between the ligature and the forceps. The extra clamp was removed after haemostasis was verified.

In both groups, the mesometrium was ligated after which the uterine body was ligated near the cervix with resorbable suture material (study group Vicryl, polyglactin 910, Ethicon, control group Maxon™, polyglyconate, Syneture). An encircling ligature was placed around the cervix. If the uterine vessels were prominent they were separately ligated.

The uterus and ovaries were removed. All the transected tissue with the attached implants or traditional sutures was finally visually inspected for haemostasis. Moreover a gauze test was performed, which involved holding haemostatic forceps and applying gauze to the transected tissue. After haemostasis was verified the abdominal wall, subcutaneous tissue and skin were closed.

### Post surgery and clinical follow-up

Daily control clinical examinations, i.e. rectal temperature, abdominal palpation and visualization of the surgical wound for dehiscence, bleeding or infection, were performed on day 1–10, until suture removal.

### Comparison of groups and larger versus smaller dogs

Weight and age of dogs as well as duration of ligation and surgery were compared. Evaluation of effect of size of dogs was performed by comparing larger dogs, defined as higher or equal to the group’s mean body weight, with smaller dogs. Time is given as minutes′ and seconds′′. The level of statistical significance was defined as P ≤ 0.05 (Student’s *t* test, two-tailed distribution, two-sample unequal variance).

### Definition of times

#### Duration of surgery was defined as from start of skin incision to last skin suture

In the control group, the definition of time for ligation was from identification of the uterine horn (start) until transection of the second ovarian pedicle. In detail, a pair of haemostatic forceps was used to clamp the ovarian pedicles and make a groove. The ovarian pedicle was single ligated and the excess suture material was cut. A haemostatic clamp was placed between the ovary and ligature with an extra clamp on the suspensory ligament after which the ovarian pedicle was cut (stop).

In the implant group, the definition of time for ligation was from identification of the uterine horn (start) until transection of the second ovarian pedicle, haemostasis was verified and the excess band was cut (stop).

## Results

The weight and age of the dogs (Table 1) did not differ between the two groups (P = 0.25 and P = 0.33, respectively). Five dogs were excluded due to abnormal haemogram (thrombocytopenia, anaemia and leukocytosis).

The ligation time of the mesovarium (Table 3) was significantly shortened (P = 0.02) using the self-locking implant versus a single encircling ligature with traditional suture (3′28′′ ± 1′05′′ and 5′29′′ ± 3′54′′, respectively). Total duration of surgery differed between the groups (P = 0.02) with a shortened duration of surgery using the self-locking implant (15′56′′ ± 2′47′′ and 20′39′′ ± 8′58′′, study group versus control group, respectively).

**Table 3:** Description of time used to ligate of the mesovarium and total duration of surgery

Groups	Ligation time	Total surgery time
Study group—LigaTie^®^	3′28′′ ± 1′05′′*	15′56′′ ± 2′47′′*
Control group—suture	5′29′′ ± 3′54′′*	20′39′′ ± 8′58′′*

Data are mean ± SD

* Values differ significantly between groups

Among the control dogs, 10 dogs had a bodyweight higher than group’s mean body weight of 12.8 kg and 14 dogs lower than the group’s mean body weight. Duration of surgery for control dogs higher and lower than the group’s mean body weight was 27′45′′ ± 9′36′′ versus 15′34′′ ± 3′24′′ (P = 0.003). The ligation time in the control dogs (suture) higher and lower than the group’s mean body weight was 7′50′′ ± 5′08′′ versus 3′49′′ ± 1′13′′ (P = 0.04).

In the implant group, 10 dogs had bodyweight higher or equal to the group’s mean body weight of 10.7 kg and 11 dogs were lower than the group’s mean body weight. Duration of surgery for the dogs in the implant group higher and lower than the group’s mean body weight was 17′43′′ ± 2′25′′ versus 14′20′′ ± 2′03′′ (P = 0.003). The ligation time in the study group higher and lower than the group’s mean body weight was 4′11′′ ± 1′08′′ versus 2′49′′ ± 0′29′′ (P = 0.004).

In a comparison in between groups of the larger dogs, bodyweight higher or equal to the groups’ mean body weights, ligation time was shorter in the implant group versus control group, (P = 0.05) and duration of surgery was shortened in the implant group (P = 0.009).

The corresponding comparison in between groups of the smaller dogs, bodyweight lower than the groups’ mean body weights, ligation time was shorter in the implant group versus control group, (P = 0.01) whereas duration of surgery did not differ (P = 0.27).

No intraoperative haemorrhage was observed in the implant group. Intraoperative haemorrhage was observed once in a dog (bodyweight 21 kg) in the control group from the left ovarian pedicle. The tissue was grasped with haemostatic forceps, an additional ligature was applied and the bleeding stopped.

In one implant it was observed the band inside the locking case was tilted, outside guiding tracks on one side of the locking case, after the loop was tightened. The locking mechanism still functioned as intended, allowed one-way motion only and held the tissue firmly compressed.

No postoperative haemorrhage was observed in any of the dogs and all dogs recovered uneventfully. No wound complications were observed and the general status of dogs in all the postoperative clinical examinations was considered normal.

## Discussion

The results of this feasibility study suggested the implant can be used to ligate the canine mesovarium. Moreover, compared with traditional single suture ligation, the results suggested that time needed to ligate the ovarian pedicle and duration of surgery were significantly reduced with the implant. Time needed to ligate the ovarian pedicle and total duration of surgery were higher for larger dogs than smaller dogs.

A previous study found that duration of a bitch spay surgery was shortened by about 15 min when cable ties were used for ligation [10, 11]. Comparisons should be made cautiously because the previous study used cable ties in mesovarium and uterine stump. Another difference was that in the previous study an applicator was used to tighten and cut excess band of the cable ties, with additional time saved compared to manual handling. In some implant dogs of the present study, time was used to create a rounded cut-off end of the band whereas no such time was used in the control group. An applicator designed to tighten and cut the excess band of the present implant may be preferable.

Ligation time was shortened when the self-locking implant was used, compared to use of a traditional suture ligation. The dogs of the control group were larger, and although body weight between groups did not differ significantly, this may have been an important contributor to the difference in duration of surgery. The difference between control and implant dogs in duration of surgery exceeded the difference in ligation time, which may be explained as an effect of body size and also lack of randomization. We chose not to clamp and compress tissue with forceps in the implant group because it could cause some haemostasis of its own and may therefore obscure an insufficient compression of the tissue. However, to compress tissue and make a groove is the recommended method of traditional ligation of the ovarian pedicle. Intraoperative haemorrhage from the mesovarium was observed in one dog in the control group, which also added to the difference in time between groups. The mesovarium was re-ligated and haemostasis was confirmed. A double ligation of the ovarian pedicle is the recommended method [27, 28], and the observed intraoperative complication in the control group suggests that the advised use of double ligation of the ovarian pedicle is justified. In total 42 ovarian pedicles were ligated with the device and no haemorrhage was observed. An important aspect of biocompatibility is the success of an implantable medical device in fulfilling its intended function [29].

Previous studies reported a higher incidence of intraoperative haemorrhage and longer surgery duration in large size and deep chested dogs [2, 3]. In the present study’s comparison of larger versus smaller dogs, duration of surgery and ligation times were longer in the larger dogs, in agreement with the previous studies.

The mechanical properties of the self-locking implant fulfilled the required ability to compress the tissue of the canine ovarian pedicle. In some implants marks were detected on the surface of the flexible band, caused by the ejector mechanism of the mould at manufacturing. In one implant we observed the band inside the case was tilted outside the guiding track after the loop was tightened. An uneven surface or band outside guiding track could negatively affect a smooth passage of the flexible band through the case and make it more difficult to tighten the loop. Increased friction at tightening of loop may be misinterpreted by the surgeon as sufficient compression of tissue and therefore constitutes a risk factor. The results confirmed the locking mechanism functioned as intended and locked the loop tight in all cases.

There were study limitations. Surgeries were not done in a random order, i.e. controls were done first and implants were done second. The difference in duration of surgery and ligation times should therefore be interpreted cautiously. Follow-up was limited to a short period, 10 days. However, postoperative haemorrhage from the ovarian pedicle is usually diagnosed during the first 24 or 48 h after surgery. The study of tissue reactions at resorption was outside stipulated aim of the present study and may be regarded as a study limitation. However, the resorbable material of the implant is well investigated and its safety is clinically established [26]. In a previous study [25], the tissue response of the implant was in agreement with the natural expected response after implantation of a resorbable medical device, a foreign body reaction, which is transient. The tissue reaction and resorption of suture polymer material is well described. After complete resorption of the material, only connective tissue will remain [29, 30].

We conclude ligation of the ovarian pedicle with the implant was feasible and the results suggested that it saved time; however further studies involving more dogs are needed.

## Abbreviations

GA: glycolide
TMC: trimethylene carbonate
GMP: good manufacturing practice
